# Exploitation of Information as a Trading Characteristic: A Causality-Based Analysis of Simulated and Financial Data

**DOI:** 10.3390/e22101139

**Published:** 2020-10-08

**Authors:** Catherine Kyrtsou, Christina Mikropoulou, Angeliki Papana

**Affiliations:** 1 Department of Economics, University of Macedonia, 54636 Thessaloniki, Greece; cmikro@uom.gr (C.M.); angeliki.papana@gmail.com (A.P.); 2 EconomiX, University of Paris 10, 92001 Paris, France

**Keywords:** information endogenization, nonlinear connectivity, direct causality, stock portfolios

## Abstract

In financial markets, information constitutes a crucial factor contributing to the evolution of the system, while the presence of heterogeneous investors ensures its flow among financial products. When nonlinear trading strategies prevail, the diffusion mechanism reacts accordingly. Under these conditions, information englobes behavioral traces of traders’ decisions and represents their actions. The resulting effect of information endogenization leads to the revision of traders’ positions and affects connectivity among assets. In an effort to investigate the computational dimensions of this effect, we first simulate multivariate systems including several scenarios of noise terms, and then we apply direct causality tests to analyze the information flow among their variables. Finally, empirical evidence is provided in real financial data.

## 1. Introduction

During the last few decades, research in finance has pointed to biases and sub-optimal decision-making processes as the drivers of investors’ behavior. Many alternatives have been proposed so as to rethink the strong assumptions of rationality and perfect information. According to Lo and Mackinlay [1] and Lo [2], economic agents’ actions can be influenced by various behavioral biases. The fact that, in terms of expectations, investors appear to be so different, since they do not share the same analytical skills, capital to invest, or even profit-maximizing goals, complicates the identification of patterns in real data. The role of heterogeneity in expectations is crucial. As demonstrated by Assenza et al. [3], in the event of a negative shock, even a small fraction of pessimistic forces coordinate and, due to positive feedback mechanisms, confidence is destroyed, leading to an expansion of market collapse. Hommes [4] presents heterogeneity and heuristics switching as detrimental factors of market dynamics. When positive feedback activates, market prices fluctuate strongly. Frijns et al. [5] relate the stylized facts observed in financial markets to an individual investor’s portfolio selection process, which is significantly driven by their risk perception, behavioral characteristics, and socio-demographic factors. As Peiro [6] shows, heterogeneity in the investment horizon may affect the significance of skewness in the portfolio construction process. In the short term, both fat tails and asymmetry contribute to the non-normality of the return distribution, while in broader horizons kurtosis seems to better explain deviations from normality. This comes in agreement with Prakash et al. [7], who suggest that the shape of a stock return distribution changes with the investment horizon.

Although information constitutes a crucial factor contributing to the evolution of financial systems, heterogeneous investors ensure the flow of information. The trading decision, strongly affected by the linear or nonlinear underlying trading strategies and filtering of news, can give birth to new information that enters the diffusion mechanism of the market and spreads rapidly. In this process, information englobes behavioral traces of traders’ decisions and represents their actions. The endogenization of information creates risk that significantly affects traders’ positions, capital flows, connectivity among assets, and finally asset allocation. 

The observed non-normal distribution of asset returns is often attributed to the dominance of irrational investors following active trading strategies. Investors whose actions are affected by anchoring and disposition effects participate in the buildup of a trend-forcing price evolution. Thurner et al. [8] show that trading strategies, characterized by leverage, lead to nonlinear positive feedback mechanisms and the amplification of price movements that generate fat tails and volatility clustering. Along with the presence of fat tails, Daniel and Moskowitz [9] and Barroso and Santa-Clara [10] relate the momentum returns with the presence of negative skewness. Jacobs et al. [11] show that overweighting left-skewed stock returns distributions and underweighting the right-skewed ones can lead to profitable momentum strategies. Ekholm and Pasternack [12] suggest that the manner of releasing positive and negative information induces skewness in the return distribution. Through simulation experiments, Wen et al. [13] provide evidence that biases, such as overconfidence and regret aversion, determine the reaction of investors to nonlinearly received information and lead to skewed and leptokurtic returns. According to Xu [14], the existence of skewness in stock returns may be the result of the invertor’s reaction to the returns themselves. As Ruttiens [15] points out, a rational investor will favor stocks presenting the highest odd moments (expected value and skewness) and the lowest event moments (variance and kurtosis). Other trading characteristics, such as trading volume and heterogeneity, seem to justify the appearance of asymmetry in returns series (Hutson et al. [16]; Albuquerque [17]). Finally, the inability to implement appropriate and effective corporate governance can be the source of positive skewness in data (Bae et al. [18]).

Despite the simplistic character of the assumption of normality, the conventional approach to build a diversified portfolio has appeal due to the ease of implementation. In this framework, practitioners need only to consider for each asset class its mean, variance, and covariances, with the latter introducing the additional restrictive hypothesis of linear relationship for each pair of asset classes. Nevertheless, in dynamically unstable markets where information canalizes traders’ characteristics into prices, diversifying asset portfolios can become a complicated procedure. To this end, research on nonlinear analysis suggests alternative techniques to the standard mean-variance framework of Markowitz [19]. Boginski et al. [20] formulate the portfolio selection problem as the maximum weight s-plex problem in the market graph. Fernandez and Gomez [21] generalize the mean-variance model by using artificial neural networks to calculate the efficient frontier. Huang [22] provides a new definition of risk by taking into consideration investors’ perceptions of the severity level of the potential loss and redefines the portfolio selection problem under this new definition. In line with the empirical evidence about the presence of heavy-tail distributions, Kraft and Steffensen [23] and Diesinger et al. [24] show that assets can be modelled as jump-diffusion processes.

As follows, the trading-based non-normality is linked to high skewness and kurtosis in asset returns and can modify drastically the portfolio (basket of variables) structure. With the aim to investigate the computational dimensions of this effect, we use multivariate systems of variables where several scenarios of disturbances are considered. Then, a set of direct causality measures is employed to analyze the information flow among the variables. In this simulation exercise, the goal is to demonstrate that non-Gaussianity in a system is able to destabilize the fundamentally defined linkages. The impact becomes more pronounced when the initial connectiveness is nonlinear, which technically may be interpreted in terms of trading activity. The empirical validation of the effect of trading information on the variables’ connectiveness is provided through an application to a concentrated and mixed five-stock portfolio.

## 2. Simulation Experiment Design

In an attempt to concretize the effect of informational signals in the sense of random disturbances on the connectivity, we use three stochastic systems. Their residual terms are defined in different ways, so that the simulated time series obey irregular characteristics. 

The two main stochastic systems, often used in the literature for the evaluation of causality measures, with Gaussian noise terms are (i) a linear vector autoregressive (VAR) of order 4 in five variables (Schelter et al. [25]) and (ii) a nonlinear VAR of order 3 in five variables with linear (X1→X3, X4↔X5) and nonlinear couplings (X1→X2, X1→X4) (Montalto et al. [26]) (hereafter, S1 and S2, respectively). In an effort to include alternative forms of nonlinearity in the construction of variables, we built a third system on the basis of S2, in which X1 is described by a noisy Mackey Glass process (Kyrtsou and Terraza [27]). To consider the effect of data length, in all cases four different samples are selected—i.e., 512, 1024, 2048, 4096.

The presence of several lags in the simulated systems as well as the diversity in the nature of the relationships help establish a direct connection with the trading practice in financial markets. The systems evolve because of the combination of value signals (in technical analysis, the indicator signals are usually expressed by an inequality in terms of past values (value signal)), since the current state of each variable depends on past information of the same or other variable. The delay in the system equations measures the speed at which imperfectly reflected information is incorporated into X_t_. Lagged information can also be transferred non-propositionally (nonlinear lagged X terms) into X_t_, revealing that each variable at time *t* either over- or under-reacts to the past. Both conditions determine the spreading of information flow and feedback within the system.

### 2.1. System S1 by Schelter et al. (2006)

The system S1 is represented by the following set of equations.

$$x_{1,t}=0.4x_{1,t-1}-0.5x_{1,t-2}+0.4x_{5,t-1}+\varepsilon_{1,t},$$

$$x_{2,t}=0.4x_{2,t-1}-0.3x_{1,t-4}+0.4x_{5,t-2}+\varepsilon_{2,t},$$

$$x_{3,t}=0.5x_{3,t-1}-0.7x_{3,t-2}-0.3x_{5,t-3}+\varepsilon_{3,t},$$

$$x_{4,t}=0.8x_{4,t-3}+0.4x_{1,t-2}+0.3x_{2,t-3}+\varepsilon_{4,t},$$

$$x_{5,t}=0.7x_{5,t-1}-0.5x_{5,t-2}-0.4x_{4,t-1}+\varepsilon_{5,t},$$

where $\varepsilon_{i,t}\sim N\left(0,1\right),i=1,\ldots ,5$.

**Figure 1:**
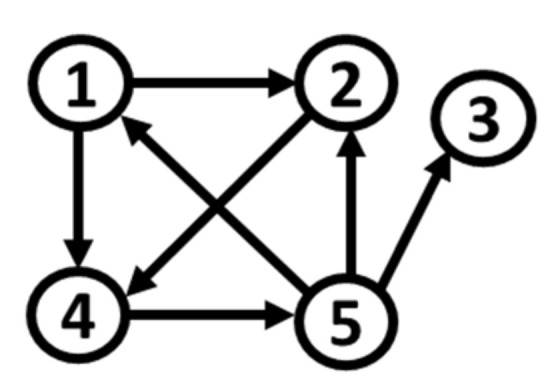
Path diagram of S1 (the arrows denote the direction of causality).

Based on S1, and by changing the distribution of noise terms $\varepsilon_{i,t},i=1,\ldots ,5$, we formulate five additional simulation systems. Their connectivity network remains intact, as shown in Figure 1. In the initial system S1, all the variables present mesokurtic and symmetric behavior.

**S1t:** S1 with noise terms $\varepsilon_{i,t},i=1,\ldots ,5$ from a *t*-Student distribution, with df = 2 degrees of freedom. The generated series exhibit leptokurtic behavior that increases with the sample. Among all the variables, X5, participating in more couplings than the rest, seems to be more sensitive to residual irregularity, having the highest kurtosis and varying skewness values.

**S1n:** S1 with noise terms $\varepsilon_{i,t},i=1,\ldots ,5$ from the following GARCH(1,1) model:

$$\varepsilon_{i,t}=\sigma_{t}w_{t},$$

$$\sigma_{t}^{2}=a_{0}+a_{1}\varepsilon_{i,t-1}^{2}+b_{1}\sigma_{t-1}^{2},$$

where $w_{t}$ is a Gaussian white noise process with $a_{0}=0.2,\;a_{1}=0.2,\;b_{1}=0.75$. The resulting time series exhibit leptokurtic and asymmetric behavior. Again, for X5, which is more affected, we obtain the highest kurtosis and positives skewness.

**S1b:** S1 with noise terms $\varepsilon_{i,t},i=1,\ldots ,5$ from a beta distribution, with parameters a=20, b=2. All the variables present abnormal negative skewness.

**S1g:** S1 with noise terms $\varepsilon_{i,t},i=1,\ldots ,5$ from a GARCH(1,1) model, as defined for S1n, where $w_{t}$ follows the gamma distribution with parameters =16, b=1/4. As in S1n, the simulated time series are leptokurtic and asymmetric. X5 has the most important kurtosis and positive asymmetry as well.

**S1f**: S1 with noise terms $\varepsilon_{i,t},i=1,\ldots ,5$ resulting from the following FIGARCH(1,d,1) model:

$$h_{t}=\omega +b\;h_{t-1}+\left\{1-b-\varphi {\left(1-L\right)}^{d}\right\}\varepsilon_{t}^{2},$$

where $\varepsilon_{t}$ is a Gaussian white noise process and ω=0.2, a=0.2,b=0.7,d=0.6. Contrary to the previous versions of S1, where fat tails are assumed in the error term, the obtained kurtosis is slightly above three, even for the largest sample. 

### 2.2. Systems S2 by Montalto et al. (2014) and S3

The system S2 is represented by the following set of equations.

$$x_{1,t}=0.95\sqrt{2}x_{1,t-1}-0.9025x_{1,t-2}+\varepsilon_{1,t},$$

$$x_{2,t}=0.5x_{1,t-2}^{2}+\varepsilon_{2,t},$$

$$x_{3,t}=-0.4x_{1,t-3}+\varepsilon_{3,t},$$

$$x_{4,t}=-0.5x_{1,t-2}^{2}+0.25\sqrt{2}x_{4,t-1}+0.25\sqrt{2}x_{5,t-1}+\varepsilon_{4,t},$$

$$x_{5,t}=-0.25\sqrt{2}x_{4,t-1}+0.25\sqrt{2}0.5x_{5,t-1}+\varepsilon_{5,t},$$

where $\varepsilon_{i,t}\sim N\left(0,1\right),i=1,\ldots ,5$.

With the aim to complicate the structure of the driving variable in S3, X1 is modelled as a noisy Mackey Glass with c = 10 and τ = 2. The remaining equations are identical to those of S2.

$$x_{1,t}=\frac{2.1\left(x_{1,t-2}\right)}{1+x_{1,t-2}^{10}}-0.05x_{1,t-1}+\varepsilon_{1,t}.$$

Although the system S2 is disturbed by normally distributed errors, as Table 1 reports, the variables X2, X4, and X5 are leptokurtic and skewed. The resulting non-normality can be explained by the amplification of the information flow towards X2 and X4, as well as by the indirect transmission to X5 via X4. In system S3, similar conclusions can be drawn only for X2 and X4. The moment statistics of X5 clearly converge to their normal distribution values. The appearance of fat tails in systems, where the nonlinear skeletons are perturbed by gaussian noise, has been studied under the term of endogenous heteroskedasticity in Kyrtsou [28] and Ashley [29].

**Figure 2:**
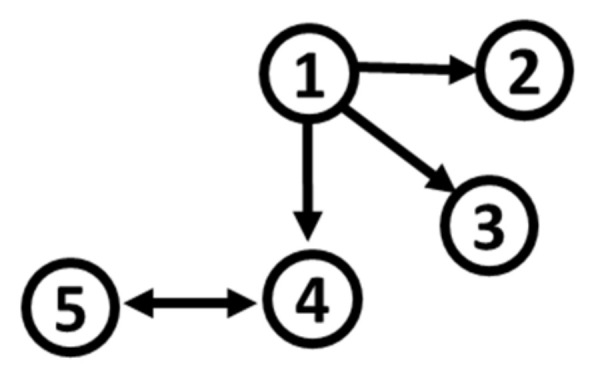
Path diagram of S2 and S3.

Based on S2 and S3, and by modifying the distribution of noise terms $\varepsilon_{i,t},i=1,\ldots ,5$, as for S1, we define five additional simulation systems. Their path diagram is represented in Figure 2. In the initial parametrization of S2, even though residuals are white noises, three (X2, X4, and X5) out of five variables obey non-normal characteristics, such as high kurtosis (around 10) and skewness (around 2, either positive or negative). For S3, two (X2 and X4) out of five variables deviate from normality, with lower values of kurtosis (around 8) and skewness (around 1, either positive or negative).

**S2t and S3t:** S2 and S3 with noise terms $\varepsilon_{i,t},i=1,\ldots ,5$ from a t-Student distribution, with df = 2 degrees of freedom. The inclusion of t-student disturbances aggravates the non-normality. More specifically, the kurtosis and skewness of variables X2, X4, and X5 double if compared with their respective behavior in the original system S2. In S3, the amplification gives birth to extreme fat-tail and asymmetric behavior for all variables. It is worth noticing that, even for small sample sizes, the kurtosis approaches the value of 250, while the skewness is about 15. This finding refines the view that the nature of shock matters a lot in the propagation mechanism within a system.

**S2n and S3n:** S2 and S3 with noise terms $\varepsilon_{i,t},i=1,\ldots ,5$ from the corresponding GARCH(1,1) model, as defined for S1n. Although all the variables are skewed and leptokurtic, the kurtosis and skewness of X2, X4, and X5 reach high values. Again, the amplification of irregularity is more pronounced for S3.

**S2b and S3b:** S2 and S3 with noise terms $\varepsilon_{i,t},i=1,\ldots ,5$ from a beta distribution, with parameters a=20, b=2, producing negative skewness. The simulated results show that the beta distribution of the noise terms is imposed on the mean structure of the system, destroying the excess kurtosis we detected for the nonlinearly connected variables X2, X4, and X5 of S2. On the contrary, in S3 the kurtosis turns into platykurtic values.

**S2g and S3g:** S2 and S3 with noise terms $\varepsilon_{i,t},i=1,\ldots ,5$ from a GARCH(1,1) model, as for S1g. The distributional characteristics of the system variables are similar to those of the system S2n. It is worth mentioning the steadily negative asymmetry of variable X4 in the case that the residuals follow a GARCH-type process. If we compare the strength of non-Gaussianity between S2 and S3, we come to the conclusion that the specific nonlinearity in the skeleton of the third system favors the detection of higher 3rd and 4th moment statistics via interaction.

**S2f and S3f**: S2 and S3 with noise terms $\varepsilon_{i,t},i=1,\ldots ,5$ from a FIGARCH(1,d,1) model, similar to S1. For both systems, clear leptokurtic behavior is detected for X2, X4, and X5, but with a lower intensity. Regarding skewness, we notice a remarkable increase as the sample size rises, with values reaching 5 and 6 (S2 and S3 respectively) for X2, as well as −5 and −6 for X4 (S2 and S3, respectively).

To provide a schematic description of the methodological part, we present the simulation experiment in four steps. First, we simulate the systems S1, S2, and S3. In the second step, we introduce irregularity in the noise terms, as described above. Then, we identify couplings using direct causality methods to capture the information flow. In the last step, performance metrics are applied to verify the consistency of the obtained results.

## 3. Connectivity Measures and Performance Metrics

After describing the systems, including both linear and nonlinear couplings, together with the irregular characteristics of the residual terms able to give rise to abnormal values for skewness and kurtosis, we apply three multivariate (direct) measures of causality, instead of bivariate ones, to better apprehend the information flow. More specifically, we intend to identify the impact of introducing non-Gaussianity in the stochastic systems S1, S2, and S3 into the connectivity among their variables. Let us consider a multivariate system with K variables, where X is the driving variable (source), Y is the response variable (target), and there also K−2 confounding variables $Z=\left\{Z_{1},\ldots Z_{K-2}\right\}$. The multivariate causality measures capture the direct causal influence from X to Y, conditioning on the remaining variables (X→Y|Z).

The Restricted Conditional Granger Causality Index (RCGCI) is an extension of the standard Conditional Granger Causality Index (Geweke [30]), including dimension reduction so that the curse of dimensionality can be effectively addressed (Siggiridou and Kugiumtzis [31]). Computationally, a modified backward-in-time selection method is selected to restrict the VAR model. The choice of the appropriate subset of lagged terms is based on the time series property—that is, the dependence structure is closely related to the temporal order of the variables. In this way, the unrestricted VAR is estimated based on the selected lagged variables. Except for the fact that the lagged terms of the driving variables are eliminated, the restricted model is similarly constructed. The RCGCI is then calculated as the logarithm of the ratio of the variances of the restricted ($\mathrm{var}\left(s_{R}^{2}\right)$) and of the unrestricted model $\mathrm{var}\left(s_{U}^{2}\right)$:

$${\mathrm{RCGCI}}_{X\to Y|Z}=\ln\frac{\mathrm{var}\left(s_{R}^{2}\right)}{\mathrm{var}\left(s_{U}^{2}\right)}.$$

The statistical significance of the RCGCI is assessed by a parametric significance test (F-statistic) on the coefficients of the lagged terms of the driving variable in the unrestricted model:

$$F=\frac{\left({\mathrm{SSE}}^{R}-{\mathrm{SSE}}^{U}\right)p_{i}}{{\mathrm{SSE}}^{U}/\left(\left(N-c\right)-P_{j}\right)},$$

where SSE is the sum of squared errors, while the superscripts U and R denote the unrestricted and restricted models, respectively. $p_{i}$ is the number of lagged components of X in the U-model for Y, c is the largest lag in the U-model, N is the data size, and $P_{j}$ is the total number of U-model coefficients.

In the Partial Mutual Information on Mixed Embedding (PMIME), the dimension reduction is effectuated via a non-uniform embedding scheme (Kugiumtzis [32]). The mixed embedding vector $w_{t}=\left[w_{t}^{X},w_{t}^{Y},w_{t}^{Z}\right]$, with varying delays from all the observed variables, is progressively built making use of a conditional mutual information (CMI) criterion. Thus, starting the process with an empty vector $w_{t}^{0}$, a new vector $w_{t}^{j}$ is formed at each step j by adding a component $w_{t}^{j}$ (from any variable), so that the future of the response variable Y, $y_{t+1}$ is best explained:

$$w_{t}^{j}={\mathrm{argmax}}_{w_{t}^{j}}\{I(y_{t+1};\;w_{t}^{j}|w_{t}^{j-1}\},$$

where I(X;Y|Z) stands for the conditional mutual information of X and Y, conditioning on the Z variables. The PMIME test in terms of conditional mutual information is expressed as follows:

$${\mathrm{PMIME}}_{X\to Y|Z}=\frac{I(y_{t+1};\;w_{t}^{Y}|w_{t}^{X},w_{t}^{Z})}{I\left(y_{t+1};w_{t}\right)}.$$

To obtain the probability densities in the estimation of the (conditional) mutual information terms, the nearest neighbors’ estimator (Kraskov et al. [33]) is employed. PMIME becomes zero in the case of no causality; otherwise, it is positive.

Respectively, the Partial Transfer Entropy on Non-Uniform Embedding (PTENUE) is introduced using the non-uniform embedding scheme (Montalto et al. [26]). Although its estimation procedure is identical to that of PMIME, an alternative nearest neighbors’ estimator of Kraskov et al. [33] is employed for computing the probability densities. The PTENUE measure is defined below:

$${\mathrm{PTENUE}}_{X\to Y|Z}=I(y_{t+1};\;w_{t}^{Y}|w_{t}).$$

The measure equals zero if causality does not exist; otherwise, it is positive. The nonlinear causality measures PMIME and PTENUE do not require a significance test. Surrogates, though, are incorporated within the estimation algorithm of the measures to form the stopping criterion regarding the mixed embedding vector. Papana et al. [34], Papana [35], and Siggiridou et al. [36,37] have shown that RCGCI, PMIME, and PTENUE outperform a large range of linear and nonlinear, bivariate and multivariate causality measures.

In the fourth step, binary classification metrics such as the sensitivity, specificity, and Matthews correlation coefficient (Tharwat [38]) are employed to evaluate the performance of the tree direct causality measures. In the simulated systems, the causality measures are estimated on the $K\left(K-1\right)$ possible pairs of variables for a system of K variables.

The sensitivity metric—i.e., the true positive rate (TPR)—quantifies the true positives (TP) against the number of real positives (P) in the data.

$$\mathrm{TPR}\;=\;\frac{\mathrm{TP}}{P}\;=\;\frac{\mathrm{TP}}{\mathrm{TP}+\mathrm{FN}}\;=\;1\;-\;\mathrm{FNR}.$$

The term true or false refers to the correct or incorrect (spurious) coupling, while positive or negative means the acceptance or rejection of couplings, respectively. If the sensitivity approaches 100%, then more correct causal links are detected over the total link detections.

The specificity metric—i.e., the true negative rate (TNR)—checks the true negatives (TN) against the number of real negatives (N) in the data.

$$\mathrm{TNR}\;=\;\frac{\mathrm{TN}}{N}\;=\;\frac{\mathrm{TN}}{\mathrm{TN}+\mathrm{FP}}\;=\;1\;-\;\mathrm{FPR}.$$

Thus, the specificity provides the percentage of rejection of spurious links over the total number of detected uncoupled cases. The percentage at which the specificity value deviates from 100% denotes the accepted spurious couplings.

Finally, the Mathews’ correlation coefficient (MCC) (Matthews [39]) is a measure of the overall performance, merging information from sensitivity and specificity by considering all the possible correct and spurious couplings, either causal or no causal. If it equals 100%, there is a perfect identification of the pairs of true and no causality.

$$\mathrm{MCC}\;=\;\frac{\mathrm{TP}\times \mathrm{TN}-\mathrm{FP}\times \mathrm{FN}}{\sqrt{\left(\mathrm{TP}+\mathrm{FP}\right)\left(\mathrm{TP}+\mathrm{FN}\right)\left(\mathrm{TN}+\mathrm{FP}\right)\left(\mathrm{TN}+\mathrm{FN}\right)}}.$$

## 4. Simulated Series Results

As reported in Table 2, the RCGI correctly identifies the connectivity network of S1 for all the samples. The performance is slightly improved as the time series length increases due to the identification of less spurious detections. The PMIME also correctly indicates connectivity. Again, similar results are obtained for all the time series lengths. However, the percentage of detecting significant causality is larger than the nominal level (5%) for the uncoupled pairs of variables. The true connectivity network of S1 is obtained with PTENUE, independent of the sample size. Less spurious cases are captured as well. On the basis of the performance metrics, the PTENUE outstands the other two measures, achieving the highest mean MCC score (96.66%) over the RCGCI (94.53%) and the PMIME (85.2%). Respectively, their mean sensitivities are very high. The difference in the performance of the measure is affected by its specificity—i.e., the true negative rate.

When a noise term from the *t*-distribution is added to S1 (S1t), the performance of RCGCI is similar to that of S1. It finds the causal links perfectly well, while the percentages of significant detections for the uncoupled pairs of variables does not exceed 5%. The performance of PMIME does not deteriorate for S1t compared to S1. The true links are detected. However, the percentage of significant detections for the uncoupled pairs of variables varies from 9.85% to 12.85%. The PTENUE performs for S1t as for S1. The true links are found, and a few spurious acceptances arise. In total, the PTENUE has the best mean performance for S1t. RCGCI comes second, with an MCC value very close to that of PTENUE.

Including the GARCH residuals in S1 (S1n) worsens the metrics of the RCGCI. The measure captures the true causal linkages, but for the uncoupled cases the percentage of significant RCGCI values varies from 8.36% to 10.38%. The PMIME gives less acceptances of spurious causalities for S1n. The best performance for the system S1n is achieved by PTENUE.

When beta-distributed errors are considered for S1b, the RCGCI finds the true connectivity network for all *n* (100%). Similar to S1, also a few spurious links are obtained. The PMIME identifies perfectly the true connections (100%) for all *n*. The uncoupled links are falsely indicated, with percentages that vary from 9.85% to 12.85%. The PTENUE indicates again the true connections, while a small number of spurious cases appear.

In the case of system S1g, the RCGCI captures the true causalities, but for the uncoupled cases the percentage of significant RCGCI values remains high (from 7.77% to 9.23%). The PMIME performs almost similarly to the RCGCI. The PTENUE continues to be the best measure. When the FIGARCH errors are considered, the PMINE puts forward more false couplings.

Table 3 and Table 4 report the results of the application to the simulated system of Montatlo at al. [26] and the new system S3. As one can see, the RCGI correctly identifies the linear relationships. On the contrary, the nonlinear links are detected with very low percentages. In addition, spurious links are indicated. On the other hand, the PMIME captures more couplings. The performance increases with the sample length. However, in terms of spurious detection the PMIME gives similar results to the RCGCI for S2, while it finds further false couplings for S3. The PTENUE performs closely to the PMIME, but in terms of the mean binary classification metrics for the system S2. However, it is clearly better than the PMIME for S3. The RCGCI achieves a pretty low mean MCC score, mainly due to its low sensitivity.

In the case of the t-distributed errors in S2, the RCGI correctly detects the true couplings. This percentage increases to 90% for the large sample. Spurious links are also revealed. This effect is more significant in the sample of 4096 observations. The PTENUE shows more true causal links, while fewer spurious relationships than the RCGCI and PMIME detect are found. In terms of performance, the PTENUE overcomes the PMIME. In the third system, the PMINE seems to be more sensitive to the nonlinearity, suggesting an increasing number of spurious couplings.

For the systems S2n, S3n, and S2g, S3g considering the GARCH residual terms, the measures produce almost identical results. The RCGI indicates the true couplings, giving comparable percentages of acceptance with S2t. The spurious detections are high, approaching 30%. The PMIME captures effectively the true connections, but at the same time it shows spurious ones. According to the sensitivity metric, the PTENUE performs as well as the PMIME, but gives fewer wrong causalities. This performance is concretized through the highest mean MCC.

Regarding S2b, the RCGCI captures efficiently both the linear and nonlinear linkages, and the percentage of spurious cases is lower than in the previously analyzed versions of the system S2. Both the PMIME and the PTENUE find the true connectivity. Nevertheless, the latter indicates a smaller number of spurious couplings. The high MCC values show that the PTENUE stands out. The significant difference between the system S3b refers to the poor performance of the RCGCI, since the number of false detections rises to 37.73%.

Finally, when the FIGARCH error term is considered in S2 and S3, the linear measure fails significantly to indicate the correct causal relationships and suggests equally high false ones. The nonlinear tools perform better, giving a lower rate of false acceptances than RCGCI for S2, except PMINE for S3, which reaches 23.93% spurious couplings.

Comparing the rate of acceptance of false causalities (specificity) between the systems S1, S2, and S3, we can conclude that more spurious couplings emerge in the nonlinear systems S2 and S3 due to the common source of driving (X1→X2, X3, X4) and the transitive indirect paths (X1→X4↔X5). It is also worth emphasizing that, among the spurious causalities, the bidirectional coupling between X2 and X4 (by definition uncoupled) is steadily detected by all measures in all samples. Therefore, we believe that the high rate of acceptance of couplings which are not derived from the initial formulation of the systems S2 and S3 indicates the creation of new structures because of the propagation.

To further deploy our rationale, we calculate the mutual information among the variables of all versions of S2 and S3. As can be seen in Table 5, when the error term obeys irregular properties that differs in intensity and nature, the dependence rises. In fact, the higher mutual information coefficient is obtained for the initially uncoupled pair X2 and X4. It occurs associatively that S2 stands out as a nonlinearly self-exciting process. According to Ocker et al. [40], in nonlinearly self-exciting processes nonlinearities impose bidirectional couplings and the structure expands. The detected spuriosity varies depending on the disturbance term that obviously affects the amplification within the nonlinear skeleton of the model. More specifically, when the noise term presents a more volatile or asymmetric profile, it imposes its own structure on the endogenous part, then amplification is braked, and the number of spurious couplings decreases.

On the contrary, in Table 6, where the driver variable X1 is generated by a noisy Mackey–Glass process, a higher level of mutual information is achieved among the variables of the system S3b. Apparently, the interaction of the beta distribution with the skeleton of S3 is spread out rapidly. In this specific model, the linear RCGCI gives around 40% spurious couplings in contrast to the nonlinear measures that identify only 10% of the false linkages.

Regardless of the sample size of the nonlinear systems under study, we observe that a significant divergence in performance between the linear and the nonlinear causality measures is associated with nonlinear relationships among the variables. In this line, for all the causality forms and disturbance terms the simulation results show that the PTENUE decodes correctly the true linkages and gives lower rates of false couplings.

## 5. Application to Real Financial Data

With the aim to provide empirical evidence about the impact of heterogeneity in information on the connectivity of variables, we built two different stock portfolios. Following the simulated systems’ construction, the first portfolio A is composed of five stocks from the French stock exchange index CAC40 to reproduce the properties of a concentrated structure. The respective listed companies are Total (FP) from the energy sector, Sanofi (SAN) from the healthcare sector, L’Oreal (OR) and Danone (BN) from the consumer defensive sector, and BNP Paribas (BNP) from the financial services sector. The required heterogeneity is achieved by considering three big capitalization stocks (FP, SAN, OR) together with two lower capitalization stocks (BN, BNP). In the second portfolio named B, the goal is to combine two different (preferably independent) concentrated structures so as to emphasize the contribution of their dynamics to the overall behavior of the portfolio. For this reason, we replace the lower capitalization stocks BN and BPN with Cipla limited (Cipla) from the healthcare sector and Britannia industries limited from the consumer defensive sector of the national stock exchange index of India NIFTY. The absence of lead-lag relationship between NIFTY and European stock indexes is pointed out by Choudhary and Singhal [41].

With a focus on considering heterogeneous investment time horizons, we select four distinct samples for each portfolio—i.e., 500, 1000, 2000, and 4000 data points—starting from the most recent observation of the dataset (i.e., 30/04/2020). The Table 7 and Table 8 report the 3rd and 4th moment statistics of all the stock returns series. The results show that the variables are highly skewed and leptokurtic. However, the maximum values of skewness and kurtosis (value in red) are obtained for different sample lengths, confirming the fact that the incorporation of historical information reveals heterogeneous aspects of investors’ activity and eventually variations in market conditions. Non-normality in the short-run subsample of 500 observations represents more speculative dimensions of trading. On the contrary, when deviations from Gaussianity become evident in longer samples, one should look at the volatile reaction of long-term-oriented investors. Deviations depend on the stock nature and do not rise or decrease proportionally with the sample size.

**Figure 3:**
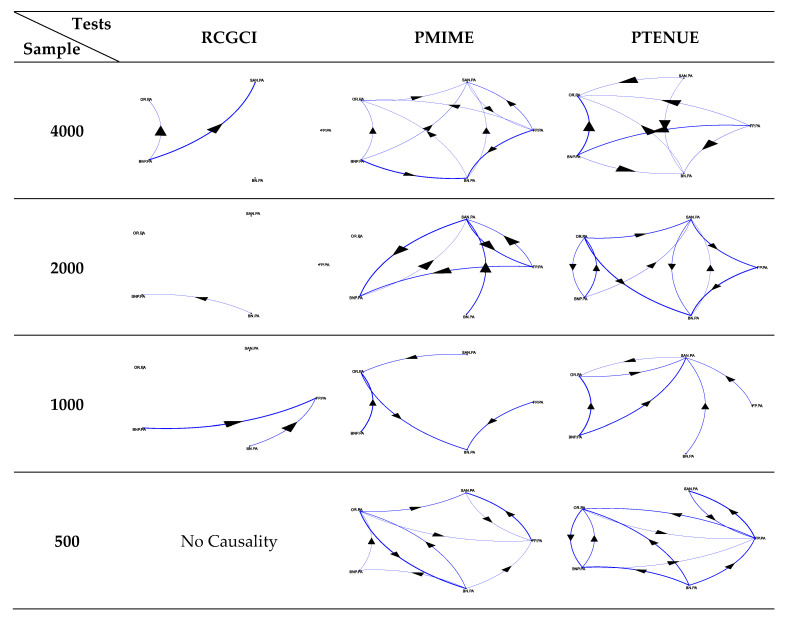
Path diagrams for portfolio A.

**Figure 4:**
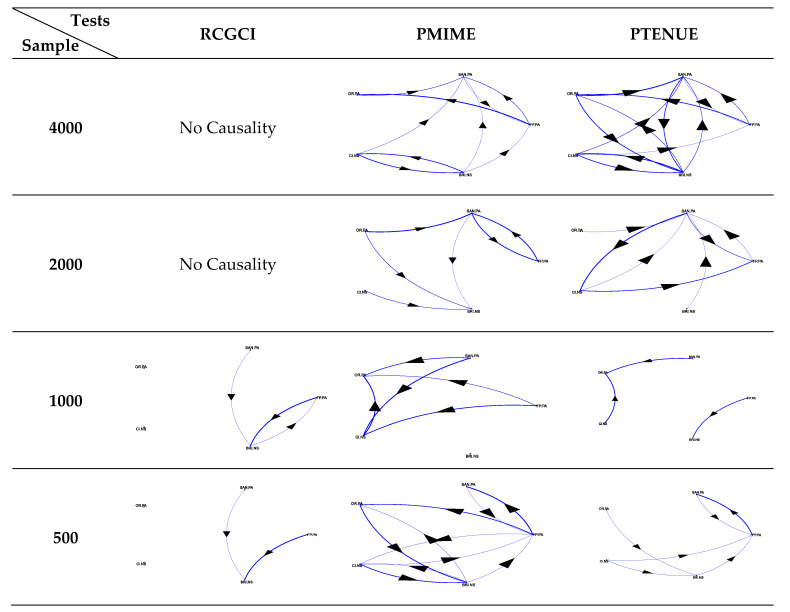
Path diagrams for portfolio B.

The application of the causality measures to the portfolios A and B helps to further illustrate the above heterogeneity. The resulting path diagrams per measure and sample are presented in Figure 3 and Figure 4. The complex interdependence between stocks is clearly indicated by the either no-causality or sparse structure captured by the linear RCGCI measure. The implementation of PMIME and PTENUE indicates rich linkages among the variables. At first glance, it seems that the strong causal forms detected by both nonlinear tools are consistent in the shorter and longer samples (i.e., 500 and 4000 points). This diversity in patterns can eventually reflect the fact that investors may be willing to take more on risk over longer periods (Andries, et al. [42]), or when the risk-free rate turns negative (Baars, et al. [43]).

Although the stock systems are composed of only five variables, the intensity of spreading for the small and large samples among the variables of portfolio A slightly differs between PMIME and PTENUE. It turns out that the combination of domestic stocks generates a nonlinearly interconnected portfolio. Of course, introducing more variables would allow richer dynamics to unfold. Therefore, in the small sample (500 obs.) of the mixed portfolio B, mixing two different sets of stocks affects the consistency of couplings and interrupts the complex pattern of portfolio A. The decrease in the standard deviation from 2.6% (portfolio A) to 1.6% (portfolio B) along with the increase in mean return from −0.106% to 0.033% is an appealing effect of the changes in connectivity over the short-term period, including the first months of the coronavirus pandemic. Respectively, the slump in the skewness value from −1.73 (portfolio A) to −0.10 (portfolio B) illustrates the tight link between the nature of stock connectivity and portfolio asymmetry that reflects the trading activity (Horwitz [44]).

Additionally, the fact that the visual representation of both portfolios is time-varying brings out the role of heterogeneity in financial markets in terms of trading horizons and the subsequent complexity of information signals. Evidence about horizon-dependent behavior has been provided by Prat [45] for the equity premium in the US stock market data over the period 1871–2008. Conclusions regarding the horizon-dependent causality between the US and the China ETF markets that increases in the long-term have been also drawn by Nie at al. [46].

Looking closer at the evolution of dependence in portfolios A and B as far as information accumulates and sample size increases will shed light on the beneficial side of the resulting dynamics. To do so, we calculate the mutual information coefficient among the stock returns. The results for the concentrated portfolio A, reported in Table 9, show that the dependence clearly intensifies as the number of observations increases. Thereby, holding stocks for a short period of time by focusing on the recent performance of the respective firms could potentially turn into a beneficial decision under favorable market conditions. When conditions deteriorate and volatility bursts, it is possible to take advantage of the nonlinear connectivity within the concentrated structures by fusing appropriately different pools of assets in an effort to address trading heterogeneity, such as the case of portfolio B.

## 6. Implications

The endogenization of information and the subsequent amplification within the system has a significant effect on variables’ connectiveness. The increasing nonlinearity and the presence of endogenous heteroskedasticity in the simulated systems S2 and S3, together with the appearance of new couplings detected as spurious by the specific statistical measures, can have several exploitable implications for the trading practice and portfolio construction. The property of nonlinearly self-exciting process applies in the set of real variables as well, where their connectivity relies upon the underlying dynamics of the data time horizon. Although, in the long run, the concentrated portfolio A and the mixed portfolio B are characterized by the rich nonlinear association of their components, in the short run mixing two different pools of stocks in portfolio B affects the connectivity and risk measurement. This finding emphasizes the importance of investors’ risk profile and time horizon in the asset allocation process.

From a broader perspective, the presence of escalating nonlinear dependences among stocks justifies the need to deal with the curse of dimensionality in financial portfolios. In complex financial markets, where the number of variables influencing the asset prices can be huge, selecting a subset able to capture market risk is an appalling challenge. Green and Hollifield [47] show that estimation errors as a result of the optimization of many assets lead to not well diversified portfolios. Nonlinear interactions among stock returns can also affect the performance of standard asset pricing models. According to Chicheportiche and Bouchaud [48], portfolios generated by nonlinear approaches outperform the Markowitz mean-variance model, while Laloux et al. [49] show that, due to the high level of noise and instability of the dependence structure, over time the use of the covariance matrix underestimates portfolio risk.

Under conditions of strong nonlinear association, a diffusion of biased information among assets can modify a portfolio’s characteristics and impact its performance. Remedies for this effect include the dynamical revising of the portfolio structure or updating asset allocation. On the other hand, exploiting informational evolution through investing in concentrated portfolios, possibly combined with style investing, can be an additional alternative. Although risky, a steady preference of individual investors for concentrated portfolios and active trading has been recorded in the literature. This is likely attributed to the existence of behavioral biases. Individual investors overestimate either the quality of their private information or their ability to interpret it (Odean [50]; Barber and Odean [51]). However, trading aggressively could also reflect their attempts to exploit superior private signals (Kyle [52]). As shown in Ivkovic et al. [53] investments made by concentrated investors can perform significantly better than the investments made by those diversifying across many stocks. Moreover, the concentration is more significant for stocks with greater information asymmetries. When the concentration increases, the risk increases nonlinearly (Horwitz [44]). Although concentrated portfolios frequently present substantial tracking errors to the benchmark, investors’ information processing is capable of transforming a theoretically suboptimal decision into a beneficial investment strategy delivering high abnormal returns (Choi et al. [54]).

Future research on the impact of connectivity among financial assets will include high-dimensional simulated systems, as well as the evaluation of real stock portfolios built under different statistical scenarios.

## Figures and Tables

**Table 1:** Kurtosis and skewness from 100 realizations of the systems S2 and S3.

S2	Statistics	X1	X2	X3	X4	X5
*n* = 512	Kurtosis	2.8503	10.5592	2.9148	10.4099	8.3788
	Skewness	−0.0035	2.3476	−0.0056	−2.3153	1.88
*n* = 1024	Kurtosis	2.9718	12.4619	2.9718	12.2474	9.8692
	Skewness	−0.0041	2.5871	0.0052	−2.542	2.0923
*n* = 2048	Kurtosis	3.0084	13.2875	3.0187	13.0753	10.514
	Skewness	0.0069	2.6586	−0.0049	−2.6187	2.1609
*n* = 4096	Kurtosis	2.996	13.3986	2.99	13.1881	10.72
	Skewness	−0.001	2.6717	−0.0006	−2.6317	2.191
**S3**						
*n* = 512	Kurtosis	2.9718	6.1201	3.0166	5.2279	3.1684
	Skewness	0.0004	1.1213	0.0169	−0.8763	0.1126
*n* = 1024	Kurtosis	2.9317	6.2288	2.9997	5.2341	3.1281
	Skewness	−0.0022	1.1212	0.0044	−0.8709	0.1167
*n* = 2048	Kurtosis	2.9323	6.0725	2.9903	5.2023	3.1374
	Skewness	0.0021	1.0896	−0.0029	−0.8581	0.1115
*n* = 4096	Kurtosis	2.9317	6.2288	2.9997	5.2341	3.1281
	Skewness	−0.0022	1.1212	0.0044	−0.8709	0.1167

**Table 2:** Outcomes from the binary classification metrics for all the S1 series. RC, PM, and PT stand for the RCGCI, PMIME, and PTENUE measures, respectively.

S1	Sensitivity			Specificity			MCC		
	RC	PM	PT	RC	PM	PT	RC	PM	PT
*n* = 512	100	99.86	99.14	93.62	88.77	97.69	92.06	86.18	96.34
*n* = 1024	100	100	100	95.77	87	97.31	94.57	84.53	96.57
*n* = 2048	100	100	100	96.15	87.54	98	95.11	85.04	97.39
*n* = 4096	100	100	100	97.15	87.38	97.15	96.36	85.04	96.32
overall	100	99.97	99.79	95.67	87.67	97.54	94.53	85.2	96.66
**S1t**									
*n* = 512	100	100	99.86	95.92	87.15	96.77	94.83	84.71	95.79
*n* = 1024	100	100	100	97.46	89.54	97.46	96.71	87.40	96.76
*n* = 2048	100	100	100	97.15	90.15	98.38	96.31	87.99	97.87
*n* = 4096	100	100	100	97.08	87.92	98	96.22	85.48	97.38
overall	100	100	99.97	96.90	88.69	97.65	96.02	86.4	96.95
**S1n**									
*n* = 512	98.71	99	99	90.38	90.92	97.92	87.20	88.01	96.49
*n* = 1024	99.71	99.86	100	90.69	92.62	98.15	88.52	90.78	97.62
*n* = 2048	99.86	100	100	91.46	90.46	98.46	89.41	88.33	98
*n* = 4096	100	100	100	89.62	94.38	99	87.49	93	98.7
overall	99.57	99.72	99.75	90.54	92.1	98.38	88.16	90.03	97.70
**S1b**									
*n* = 512	100	100	100	95.92	87.15	96.77	94.83	84.71	95.79
*n* = 1024	100	100	100	97.46	89.54	97.46	96.71	87.40	96.76
*n* = 2048	100	100	100	97.15	90.15	98.38	96.31	87.99	97.87
*n* = 4096	100	100	100	97.08	87.92	98	96.22	85.48	97.38
overall	100	100	100	96.90	88.69	97.65	96.02	86.4	96.95
**S1g**									
*n* = 512	99.14	99.43	99.71	90.85	90.77	98.23	88.08	88.21	97.48
*n* = 1024	99.57	100	100	91.54	91.54	98.38	89.24	89.56	97.89
*n* = 2048	100	100	100	92.23	92.54	98.15	90.28	90.69	97.61
*n* = 4096	100	100	100	90.77	93.69	99.31	88.77	92.13	99.41
overall	99.68	99.86	99.93	91.35	92.14	98.52	89.09	90.15	98.1
**S1f**									
*n* = 512	96.43	92.43	88.86	92.85	84.46	94.92	87.95	75.09	84.69
*n* = 1024	99.14	92.29	94.92	93.77	87.23	95.31	91.49	81.63	89.81
*n* = 2048	99.71	98.86	98	94.46	85.62	96.54	92.88	81.98	94.06
*n* = 4096	100	99.86	99.43	93.85	87.23	96.08	92.25	84.65	94.54
overall	98.82	95.86	95.30	93.73	86.14	95.71	91.14	80.84	90.78

**Table 3:** Outcomes from the binary classification metrics for all S2 series. RC, PM, and PT stand for the RCGCI, PMIME, and PTENUE measures, respectively.

S2	Sensitivity			Specificity			MCC		
	RC	PM	PT	RC	PM	PT	RC	PM	PT
*n* = 512	54.4	82.2	81.4	84.73	89.8	93.6	38.98	70.42	76.02
*n* = 1024	59.6	90.4	86.8	82.87	88.73	90.6	41.16	75.29	74.96
*n* = 2048	67.4	99.8	99.4	82.2	85.6	85.2	46.9	78.3	76.88
*n* = 4096	66	100	100	79.8	84.07	85.27	42.76	76.36	77.35
overall	61.85	93.1	91.9	82.4	87.05	88.67	42.45	75.09	76.30
**S2t**									
*n* = 512	73.2	86.2	84.4	78	88.53	93.4	47.26	71.05	77.17
*n* = 1024	80.2	88.2	90.4	73.4	87.6	92	48.06	71.42	79.23
*n* = 2048	84.2	88.8	93	72.93	88.4	91.73	51.01	72.72	80.7
*n* = 4096	90	90	96.8	69.93	89.27	91.47	52.59	74.85	83.21
overall	81.9	88.3	91.15	73.5	88.45	92.15	49.73	72.51	80.08
**S2n**									
*n* = 512	72.8	94.2	93	76.47	88.47	95.53	45.58	77.68	87.41
*n* = 1024	80.4	98.2	97.8	74.67	90.8	95.67	49.92	84.04	91
*n* = 2048	83.4	99.6	99.6	69	90.87	95.2	46.85	84.98	91.64
*n* = 4096	88.6	100	100	64.67	90.2	94.67	47.13	84.22	91.03
overall	81.3	98	97.6	71.2	90.09	95.27	47.37	82.73	90.27
**S2b**									
*n* = 512	95.2	95.2	95.8	90.6	94.6	98.33	81.67	87.45	94.23
*n* = 1024	99.6	100	100	93.27	96.6	98.47	88.75	94.33	97.35
*n* = 2048	100	100	100	90.4	95.07	98.53	84.5	91.72	97.43
*n* = 4096	100	100	100	87.2	94.33	97.93	80.16	90.58	96.41
overall	98.7	98.8	98.95	90.37	95.15	98.32	83.77	91.02	96.36
**S2g**									
*n* = 512	74.6	95.6	92.2	75.4	89.13	96	45.78	79.46	87.64
*n* = 1024	77.2	99.6	97.8	74.8	89.87	94.13	47.29	83.75	88.75
*n* = 2048	84.4	99.6	99.8	70.07	89.13	94.73	48.7	82.58	90.9
*n* = 4096	87	100	100	66.13	89.13	94.2	47.08	82.63	90.29
overall	80.8	98.7	97.45	71.6	89.32	94.77	47.21	82.11	89.4
**S2f**									
*n* = 512	60.20	87.2	85.6	84	88.47	92.6	43.58	71.96	76.79
*n* = 1024	59.80	91.4	92.2	82.67	86.8	91.53	41.29	73.17	80.34
*n* = 2048	64.80	95.2	95.4	80.27	87	89.67	42.94	76.24	79.88
*n* = 4096	70.80	96	98.2	78.93	86.6	89.13	46.02	76.14	81.52
overall	63.9	92.45	92.85	81.47	87.22	90.73	43.46	74.38	79.63

**Table 4:** Outcomes from the binary classification metrics for all S3 series. RC, PM, and PT stand for the RCGCI, PMIME, and PTENUE measures, respectively.

S3	Sensitivity			Specificity			MCC		
	RC	PM	PT	RC	PM	PT	RC	PM	PT
*n* = 512	65.4	100	99.8	93.07	80.33	94.47	62.88	72.74	90.89
*n* = 1024	63.8	100	100	92.87	78.93	95.33	61.4	70.80	94.02
*n* = 2048	64.8	100	100	93.4	81.13	95.13	62.78	73.61	93.84
*n* = 4096	62.8	100	100	91.2	80.13	94.47	56.9	72.25	92.91
overall	64.2	100	99.95	92.64	80.13	94.85	60.99	72.35	92.92
**S3t**									
*n* = 512	87	98.6	99.2	89	88.73	97.13	72.68	81.56	94.56
*n* = 1024	89	99.8	99.8	90.73	87.4	96.67	76.95	80.77	94.25
*n* = 2048	93.4	100	100	92.53	85.87	96.47	82.71	78.63	94.17
*n* = 4096	96.2	100	100	93.33	87.6	96.73	86.16	80.94	94.6
overall	91.4	99.6	99.75	91.39	87.4	96.75	79.63	80.48	94.4
**S3n**									
*n* = 512	81	100	100	85.8	85.4	96	64.62	78.17	93.23
*n* = 1024	87.4	100	100	78.13	87.53	96.4	60.14	80.83	93.98
*n* = 2048	90.4	100	100	75.6	90.27	96.53	59.77	84.66	94.07
*n* = 4096	94.8	100	100	67.33	89.53	97	54.87	83.48	95
overall	88.4	100	100	76.72	88.18	96.48	59.85	81.79	94.07
**S3b**									
*n* = 512	100	93	84.6	73.07	92.47	94.13	64.03	82.2	83.68
*n* = 1024	100	100	99.4	68.67	91.2	93.87	59.74	85.64	91.43
*n* = 2048	100	100	100	65.73	90.60	95.8	57.13	84.94	92.68
*n* = 4096	100	100	100	62.27	90.73	95.13	54.24	85.01	91.58
overall	100	98.25	96	67.44	91.25	94.73	58.79	84.45	89.84
**S3g**									
*n* = 512	82	100	99.8	84.13	87.2	94.67	63.64	80.66	91.07
*n* = 1024	89.4	100	100	77.2	86.4	96.53	61.47	79.56	94.27
*n* = 2048	94	100	100	73.93	89.13	96.8	60.78	83.21	94.58
*n* = 4096	96.6	100	100	67.73	89.6	96.6	56.91	83.74	94.25
overall	90.5	100	99.95	75.75	88.08	96.15	60.7	81.79	93.54
**S3f**									
*n* = 512	71	95.8	91.8	87	81.6	93.87	58.16	70.82	84.04
*n* = 1024	73.2	97.6	96	83.33	78.8	93.8	53.81	68.76	87.12
*n* = 2048	76.6	99.2	98.8	80.60	76.93	90.67	53.82	67.76	84.34
*n* = 4096	78.8	99.6	99.8	77.13	76.07	87.6	50.86	67.46	80.73
overall	74.9	98.05	96.6	82.02	78.35	91.49	54.16	68.7	84.06

**Table 5:** Mutual information between the variables of various S2 systems for *n* = 4096 (the values for the respective systems are denoted in different colors).

S2, S2t, S2n, S2b, S2g, S2f	X1	X2	X3	X4	X5
**X1**	-	0.0000 0.0044 0.0294 0.0000 0.0344 0.0099	0.0990 0.1366 0.1911 0.1280 0.1830 0.1923	0.0053 0.0203 0.0824 0.0000 0.0957 0.0575	0.0973 0.1734 0.2493 0.4293 0.2699 0.1980
**X2**		-	0.0280 0.0205 0.0568 0.1831 0.0535 0.0405	0.7509 0.9407 0.7874 1.2848 0.7892 1.0911	0.0818 0.1679 0.2241 0.0536 0.2179 0.2297
**X3**			-	0.0571 0.1070 0.1079 0.2644 0.1160 0.1494	0.1020 0.1691 0.1870 0.3169 0.1958 0.3289
**X4**				-	0.1306 0.2648 0.3131 0.0958 0.3152 0.3562
**X5**					-

**Table 6:** Mutual information between the variables of the various S3 systems for *n* = 4096 (the values for the respective systems are denoted in different colors).

S3, S3t, S3n, S3b, S3g, S3f	X1	X2	X3	X4	X5
**X1**	-	0.0111 0.0059 0.0232 0.7988 0.0269 0.0039	0.0022 0.0000 0.0140 0.3140 0.0054 0.0000	0.0052 0.0000 0.0259 0.9428 0.0206 0.0148	0.0000 0.0000 0.0177 0.6588 0.0104 0.0205
**X2**		-	0.0120 0.0053 0.0072 0.2340 0.0000 0.0000	0.1329 0.2757 0.2330 1.1917 0.2524 0.6074	0.0034 0.0000 0.0301 0.6000 0.0401 0.0350
**X3**			-	0.0040 0.0020 0.0113 0.7189 0.0102 0.0005	0.0024 0.0024 0.0159 0.5489 0.0116 0.0126
**X4**				-	0.0000 0.0734 0.0548 1.0924 0.0555 0.1048
**X5**					-

**Table 7:** Kurtosis and skewness of the stock returns in portfolio A.

*n* = 4000 14/9/2004–30/04/2020	FP	SAN	OR	BNP	BN
Kurtosis	16.8693	8.9997	8.8949	12.7080	7.8091
Skewness	−0.3475	−0.1641	0.2020	−0.0539	−0.1539
***n* = 2000** **21/06/2012–30/04/2020**					
Kurtosis	23.7631	7.1009	6.8242	13.3001	7.8363
Skewness	−1.2257	−0.4148	0.1141	−0.9696	−0.3779
***n* = 1000** **02/06/2016–30/04/2020**					
Kurtosis	36.1905	7.3303	10.1418	19.8451	11.8267
Skewness	−2.0231	−0.1469	0.1186	−1.9065	−0.8756
***n* = 500** **17/05/2018–30/04/2020**					
Kurtosis	28.1107	7.3561	9.4350	13.3472	13.4247
Skewness	−1.9083	−0.3948	0.1157	−1.5374	−1.2417

**Table 8:** Kurtosis and skewness of the stock returns in portfolio B.

*n* = 4000 14/9/2004–30/04/2020	FP	SAN	OR	CILPA	BRITANNIA
Kurtosis	16.8693	8.9997	8.8949	7.9616	23.1883
Skewness	−0.3475	−0.1641	0.2020	0.0052	1.7592
***n* = 2000** **21/06/2012–30/04/2020**					
Kurtosis	23.7631	7.1009	6.8242	7.9316	11.9245
Skewness	−1.2257	−0.4148	0.1141	0.4215	0.4737
***n* = 1000** **02/06/2016–30/04/2020**					
Kurtosis	36.1905	7.3303	10.1418	9.5737	14.8812
Skewness	−2.0231	−0.1469	0.1186	0.8919	0.1096
***n* = 500** **17/05/2018–30/04/2020**					
Kurtosis	28.1107	7.3561	9.4350	9.5488	14.1208
Skewness	−1.9083	−0.3948	0.1157	1.0137	−0.0525

**Table 9:** Mutual information between the stock returns of portfolios A and B.

Portfolio A	Samples	FP	SAN	OR	BNP	BN
**FP**	4000	-	0.1659	0.1928	0.2254	0.1456
	2000	-	0.1628	0.1552	0.2257	0.1413
	1000	-	0.0499	0.0865	0.1816	0.0711
	500	-	0.0555	0.0785	0.2075	0.0737
**SAN**	4000		-	0.2008	0.1463	0.1781
	2000		-	0.2295	0.1385	0.2169
	1000		-	0.1139	0.0185	0.1143
	500		-	0.1251	0.0129	0.1193
**OR**	4000			-	0.1285	0.2847
	2000			-	0.1171	0.3382
	1000			-	0.0685	0.2455
	500			-	0.0784	0.2089
**BNP**	4000				-	0.1131
	2000				-	0.1087
	1000				-	0.0469
	500				-	0.0535
**BN**	4000					-
	2000					-
	1000					-
	500					-
**Portfolio B**	**Samples**	**FP**	**SAN**	**OR**	**CIPLA**	**BRITANNIA**
**FP**	4000	-	0.1659	0.1928	0.0171	0.0044
	2000	-	0.1628	0.1552	0.2257	0.1413
	1000	-	0.0499	0.0865	0.0000	0.0133
	500	-	0.0555	0.0785	0.0177	0.0246
**SAN**	4000		-	0.2008	0.0154	0.0085
	2000		-	0.2295	0.1385	0.2169
	1000		-	0.1139	0.0006	0.0043
	500		-	0.1251	0.0174	0.0124
**OR**	4000			-	0.0082	0.0138
	2000			-	0.1171	0.3382
	1000			-	0.0022	0.0006
	500			-	0.0216	0.0027
**CIPLA**	4000					0.5459
	2000					0.1087
	1000					0.5022
	500					0.5532
**BRITANNIA**	4000					-
	2000					-
	1000					-
	500					-
